# Capillary wave tweezer

**DOI:** 10.1038/s41598-024-63154-0

**Published:** 2024-05-30

**Authors:** Bethany Orme, Hamdi Torun, Matthew Unthank, Yong-Qing Fu, Bethan Ford, Prashant Agrawal

**Affiliations:** 1 Smart Materials and Surfaces Laboratory, Faculty of Engineering and Environment, Northumbria University, Newcastle upon Tyne, NE1 8ST UK; 2 Department of Applied Sciences, Northumbria University, Newcastle upon Tyne, NE1 8ST UK

**Keywords:** Capillary, Streaming, Acoustic, Vibration, Microparticles, Mechanical engineering, Applied physics, Fluid dynamics

## Abstract

Precise control of microparticle movement is crucial in high throughput processing for various applications in scalable manufacturing, such as particle monolayer assembly and 3D bio-printing. Current techniques using acoustic, electrical and optical methods offer precise manipulation advantages, but their scalability is restricted due to issues such as, high input powers and complex fabrication and operation processes. In this work, we introduce the concept of capillary wave tweezers, where mm-scale capillary wave fields are dynamically manipulated to control the position of microparticles in a liquid volume. Capillary waves are generated in an open liquid volume using low frequency vibrations (in the range of 10–100 Hz) to trap particles underneath the nodes of the capillary waves. By shifting the displacement nodes of the waves, the trapped particles are precisely displaced. Using analytical and numerical models, we identify conditions under which a stable control over particle motion is achieved. By showcasing the ability to dynamically control the movement of microparticles, our concept offers a simple and high throughput method to manipulate particles in open systems.

## Introduction

Since the detection of optical gradient forces on particles in 1970 and invention of optical tweezers^1,2^, trapping and manipulation of micro- and nano-particles^3^ have become critical for applications including sensing^4^, molecular characterisation^5^, bio-analytics^6,7^ and 3D assembly^8,9^. Optical tweezers rely on highly focused laser beams, which can produce sufficient optical radiation pressures to trap and manipulate microparticles, nano-particles and atoms^10^. However, the excessive heat generated due to these optical gradients makes it unfavorable to be used for sensitive active particles like biological cells. Therefore, methods using electric^11–15^, magnetic^16–18^, acoustic fields^19,20^ and hydrodynamic fields have been developed to trap microparticles in gradient fields. Each mechanism offers different benefits and limitations. For example, although electric and magnetic fields offer precise manipulation, the need for particles to be polarisable or paramagnetic, results in limited application. In hydrodynamic methods, particles can be trapped in stagnation points and manipulated by controlling flow rates in opposing directions in a channel^21–25^. However, their application is limited due to the inflexibility caused by the dependence on channel design.

Acoustic tweezer platforms offer a promising method for non-invasive and label free particle manipulation. Acoustic fields rely on second order effects: acoustic radiation pressure and acoustic streaming to trap and manipulate particles. Acoustic radiation pressure based tweezers, termed as radiation force tweezers, use bulk acoustic waves^26,27^, counter-propagating traveling^28,29^ or standing acoustic waves^30,31^, and focused acoustic beams^32^ to trap particles in pressure nodes or antinodes, depending on their density and compressibility^33^. For example, standing acoustic waves are generated by the superposition of two counter-propagating waves to create a grid of antinodes and nodes^34^. By changing the phase difference between the two propagating waves, particle positions can be altered^35^. Similarly, counter-propagating traveling surface acoustic waves of different frequencies create pressure field traps, which can be modulated using the wave amplitudes^36^. Acoustic vortex based tweezers use the same concept as optical tweezers of localising the acoustic radiation pressure via focused acoustic beams^37,38^. Acoustic streaming based tweezers utilise the time-averaged second-order flow vortices, termed as acoustic streaming^39,40^ to trap and manipulate particles^41–43^. The interaction of the bulk acoustic waves with the liquid domain boundaries generates significant spatial gradients in the velocity field, due to a small boundary layer thickness^44^. This significant liquid momentum flux near the domain boundaries is observed as time-averaged, vortical flow in the liquid bulk^45,46^. These vortices can either be generated from bulk acoustic fields^47^ or from vibrating interfaces^48,49^.

While acoustic fields offer a versatile platform for dynamic micrometric-scale particle manipulation, ability to manipulate large particle volumes^50^, challenges with particle viability due to high shear forces^51^ and complex fabrication processes, still remain. Additionally, applications of open microfluidic systems for particle probing and cell analysis, and for scalable manufacturing, such as, 3D bio-printing and monolayer assemblies require programmable and mm-scale systems to control particle movement and position^52–55^.

To address these operational scaling challenges, low frequency vibrations using capillary waves offer a promising solution for collecting sub-micron sized particles at millimetric scales^56,57^. As opposed to acoustic systems, particle aggregation in the frequency range of 10–500 $\hbox {Hz}$ is a consequence of first order vibration effects^58,59^. In this work, we show for the first time, that these capillary waves can be used as programmable tweezers to trap a cluster of microparticles and dynamically translate them within a liquid volume. We show that the particle position can be dynamically altered by modulating the capillary wavelength or the displacement of the capillary wave. We characterise the movement of particles at different actuation amplitudes and the speed of capillary wave displacement and identify the optimum conditions to obtain a stable control over particle movement. Furthermore, we support our experimental observations with analytical and numerical models.

## Theoretical background

When a particle is placed in an oscillating fluid field ($u_f=u_0 \sin (\omega t)$), it experiences a drag force from the fluid motion and oscillates around a point with a phase lag, $\phi$, in its motion due to its inertia ($u_p=u_{p0} \sin (\omega t+\phi )$), given by Eq. (1)^60^.

1 $$\begin{aligned} \tan \phi = -\frac{2\rho _p r^2 \omega }{9\mu }. \end{aligned}$$

**Figure 1:**
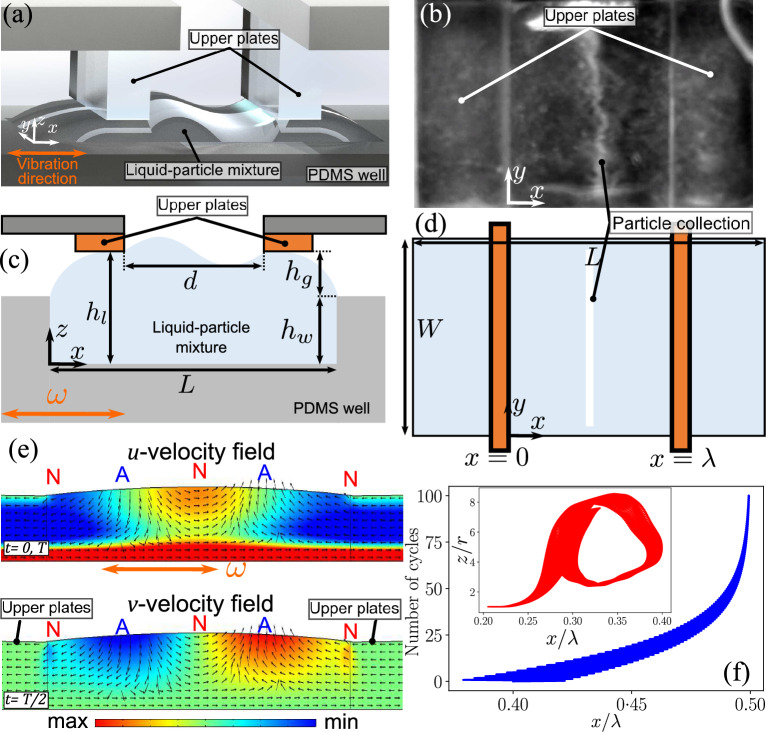
(**a**) Depiction of the experimental setup. A capillary wave is generated between the upper parallel plates by vibrating the PDMS well in the horizontal (*x*) direction. (**b**) Top view of the experiment which shows the orientation of the upper plates and the particle collection in the PDMS well. (**c**) Side view depiction of the experimental setup, where the upper plates with a separation distance (*d*) are placed on top of the liquid-particle mixture contained within the PDMS well of fixed depth ($h_w$) with a gap ($h_g$) between the well plate and parallel plates; the total height of the liquid volume is $h_l=h_g+h_w$. (**d**) Top view depiction of the experimental setup indicating the length ($L=$8 $\hbox {mm}$) and width ($W=$3 $\hbox {mm}$) of the PDMS well. (**e**) The velocity fields in the *x*- and *y*-directions (*u* and *v* velocity fields, respectively) obtained via simulations. The base is vibrated such that a wave of 1 wavelength is setup. The color map indicates the strength of the fields, the arrows indicate the direction of the fields. ‘N’ and ‘A’ represent the displacement nodes and antinodes, respectively, of the standing capillary wave. (**f**) Particle trajectory simulation in the flow fields, showing an asymptotic convergence to $x=\lambda /2$ over multiple cycles. Inset shows an example of the *z* and *x* position of a particle trapped in a streaming field; *r* is the particle radius.

Here, $\rho _p$ is the particle density, *r* is the particle radius, $\omega$ ﻿is the actuation frequency in rad/s and $\mu$ is the liquid’s viscosity. When a spatial gradient is introduced in this time-periodic flow field, such as via a capillary wave (Fig. 1e), the particles undergo displacement towards positions of lowest velocity magnitudes and collect stably (Fig. 1f)^56^. At low actuation frequencies (of the order of 10–100 Hz), this migration of a particle is due to its inertial lag ($\phi$), with the fluid motion. Due to this lag, the particle experiences flow fields of different magnitudes at different times in the oscillation cycle. As a result, the particle undergoes a displacement to the region of slower velocity fields (Supplementary information Fig. 4a) over multiple cycles^56,58^.

In our experiments, where the substrate is vibrated horizontally (along *x*-direction in Fig. 1a) to generate a capillary wave, the capillary wave field and Stokes flow (due to the oscillation of the base) superimpose resulting in a flow field with a minimum *u*-velocity magnitude, at a given *z*, underneath the capillary wave nodes (Supplementary information Fig. 3a)^61,62^. Therefore, the particles migrate to a stable collection position underneath the nodes of the capillary wave, as shown in Fig. 1b and depicted in Fig. 1d. When a wave of 1 wavelength is actuated, the nodal locations are at the center of the wave and at the pinning points (as depicted in Fig. 1c). It is important to note here that this inertial migration is induced by the drag force as opposed to inertial lift forces reported with inertial microfluidic flows at high Reynolds number^63,64^.

During these oscillations a second order flow field is also generated due to liquid inertia. Although the magnitude of this second order flow field is significantly smaller than the first order field, its time-averaged effects are observed as vortices known as streaming flows^44,56^. These streaming flows disrupt particle collection and drag them into the bulk of the liquid (inset in Fig. 1f). For a given actuation frequency (and wavelength) and amplitude there exists a ‘cut-off’ particle radius and density. Any particles with radius and density above these cut-off values will collect stably while the ones with lower values remain swirling in the liquid bulk^56^. A unique feature of such a low-frequency capillary wave system is that the cut-off particle radius and density depend on the actuation amplitude, which can be easily controlled by the user. Such an amplitude dependence of cut-off values is not possible in high frequency acoustic systems where the frequency or the device needs to be changed to alter the cut-off properties^65^.

## Results

The basic requirement of a capillary tweezer system is to induce a relative translation between a capillary wave and bulk liquid volume containing the particles. In our experimental configuration as shown in Fig. 1a, the PDMS well containing the water-particle mixture is vibrated to generate a capillary wave between two parallel plates (named as ’Upper plates’ in Fig. 1a–c). The contact line of water is pinned on the edge of the plates, where the distance *d* between the plates determines the wavelength of the wave, and thus, the actuation frequency. These upper plates are mounted on a motorised translation stage for a controlled translation of the capillary wave.

**Figure 2:**
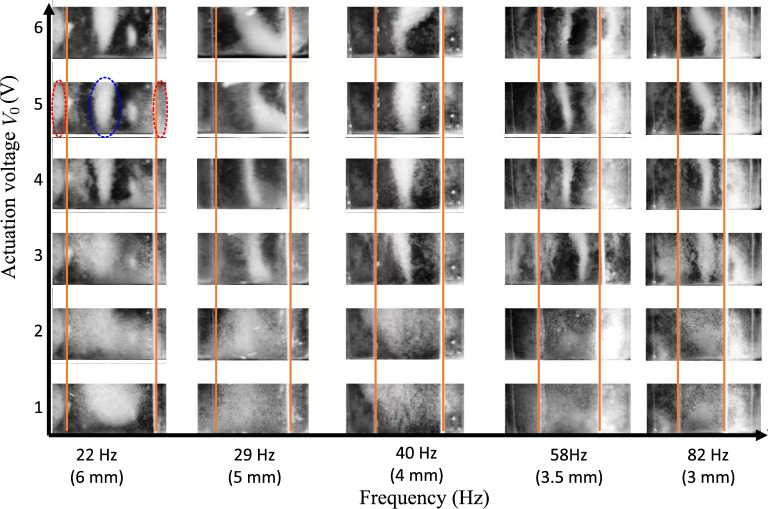
Collection of melamine resin particles ($r=$5 $\upmu \hbox {m}$) under the action of capillary wave at different amplitudes and plate separations (*d*), corresponding to different actuation frequencies and a liquid height $h_l=$0.75 $\hbox {mm}$. The orange lines indicate the edge of the upper plates or the two nodes of the capillary wave. In the image with $d=$6 $\hbox {mm}$ and $V_0=$5 $\hbox {V}$, the blue dashed line represents the particles collected stably underneath the central capillary wave node and the red dashed line represents the the particles drawn underneath the upper plates.

### Particle tweezing

Dynamic control over particle position, or particle tweezing, can be achieved via two methods, i.e., (1) capillary wave modulation (at different vibration frequency) and (2) capillary wave displacement (at same vibration frequency).

#### Capillary wave modulation

Figure 2 shows particle collection at different frequencies and actuation amplitudes. In all these cases, a capillary wave of one wavelength is actuated between the two upper plates, i.e., $\lambda =d$. By changing the plate separation distance *d*, the natural frequency of the wave can be changed through Eq. (2).

2 $$\begin{aligned} \omega ^2=(gk+\gamma k^3/\rho _l)\tanh (kh_l), \end{aligned}$$

where *g* is the acceleration due to gravity, $k=2\pi /\lambda$ is the wavenumber, $\lambda$ is the wavelength of the wave, $\gamma$ is the surface tension and $\rho _l$ is the density of the liquid. Particle collection at low amplitudes is hardly observed at all the explored frequencies within one minute of the experimental run-time. This run-time restriction is placed to reduce the effect of evaporation, which can alter the resonant frequency or lead to liquid dry-out in the given ambient conditions (temperature: 18 ${}^{\circ }\,\hbox {C}$ to 25 ${}^{\circ }\,\hbox {C}$ and relative humidity 25% to 40%). At these amplitudes, either the collection forces on the particles are not high enough to displace the particles from their initial deposited state, or the collection timescales are larger than the experiment run-time.

As the actuation amplitude $V_0$ is increased, particles collect in a single line underneath the central node of the capillary wave (Fig. 2 and Supplementary video SV1). By dynamically changing the position of one of the upper plates relative to the other, the wavelength of the wave can be changed, which changes the position of the particles dynamically. Some particles are observed to collect underneath the edge of the upper plates as those two locations also constitute the nodes of the capillary wave. The positions $x=0$, $\lambda /2$ and $\lambda$ form a stable collection position, whereas $x=\lambda /4$ and $3\lambda /4$ form an unstable region, as shown in Supplementary information Fig. 4b. Therefore, any particles that are present between the positions [$\lambda /4$, $\lambda /2$] and [$\lambda /2$, $3\lambda /4$] are collected stably at $x=\lambda /2$, while those present between [0, $\lambda /4$] and [$3\lambda /4$, $\lambda$] are collected stably at $x=0$ and $x=\lambda$, respectively. As the amplitude is further increased, the second order streaming forces start affecting particle collection, which drags particles away from their stable collection location (Supplementary video SV1).

#### Capillary wave displacement

**Figure 3:**
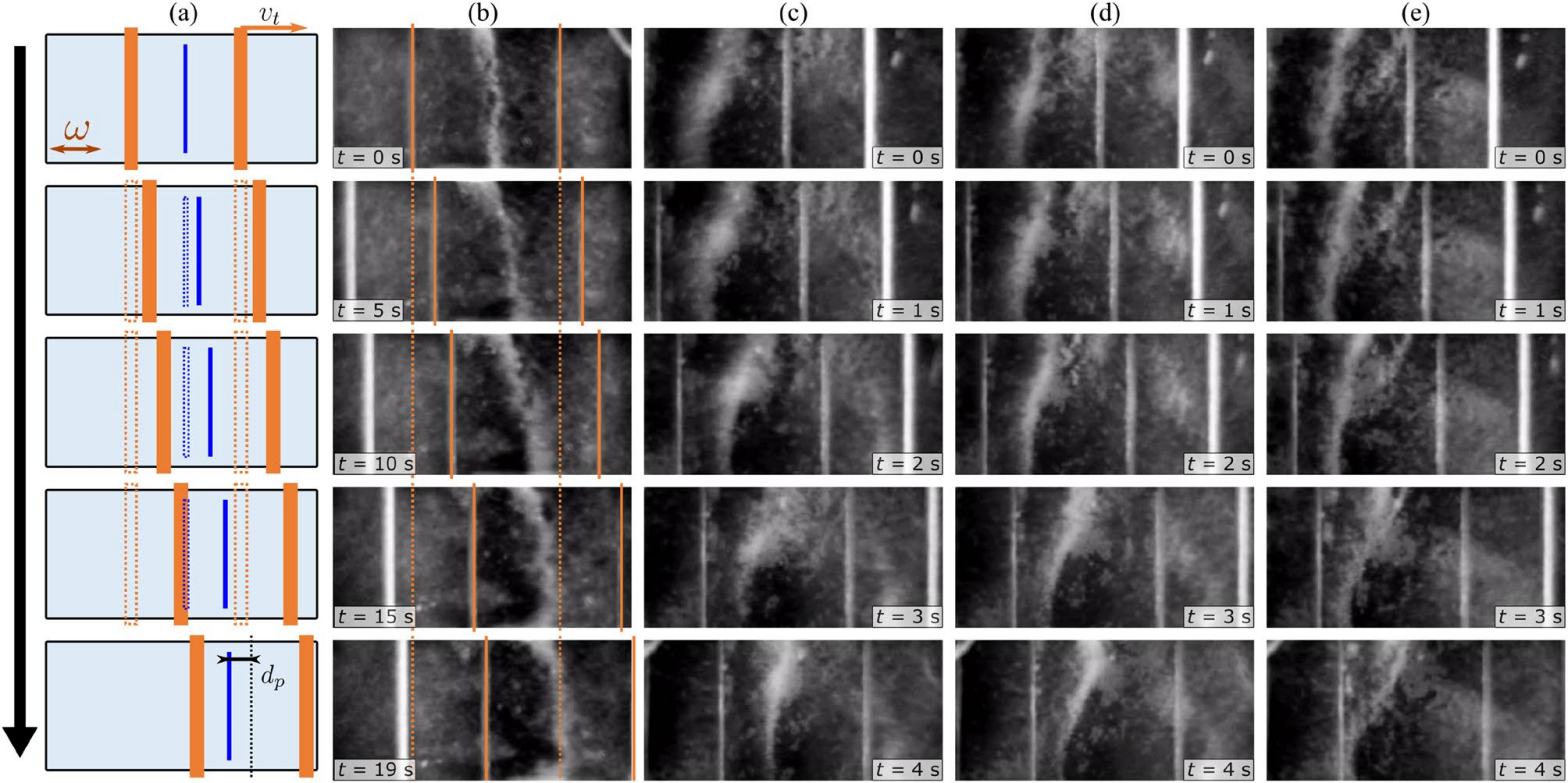
Control of particle position using capillary tweezers. (**a**) Schematic drawing of the capillary wave and particle displacement. The current position of the upper plates is indicated by the solid orange lines while the dashed orange lines indicate their original positions. The blue solid line indicates the current position of the particles, while the dashed blue line indicates their original position. (**b**) Experimental sequence of particle displacement at a translation speed of $v_t=$ 0.1 $\hbox {mms}^{-1}$ at an actuation amplitude of $V_0=$ 3 $\hbox {V}$. (**c**) $v_t=$ 0.5 $\hbox {mms}^{-1}$ and actuation amplitude of $V_0=$ 6 $\hbox {V}$. (**d**) $v_t=$ 0.5 $\hbox {mms}^{-1}$ and actuation amplitude of $V_0=$ 4 $\hbox {V}$. (e) $v_t=$ 0.5 $\hbox {mms}^{-1}$ and actuation amplitude of $V_0=$3 $\hbox {V}$. In all the experiments, the upper plate separation distance $d=$ 3.5 $\hbox {mm}$, which corresponds to a frequency of $f=$ 58 $\hbox {Hz}$ for a capillary wave of one wavelength. The liquid height $h_l=$ 0.5 $\hbox {mm}$.

Another method to achieve control over particle position and movement is by dynamically moving the two upper plates simultaneously, i.e, displacing the capillary wave without changing the wavelength or frequency (Fig. 3 and Supplementary video SV2). As particles are trapped in a stable equilibrium underneath the central node of the capillary wave, the particles undergo translation with the upper plates (Fig. 3).

**Figure 4:**
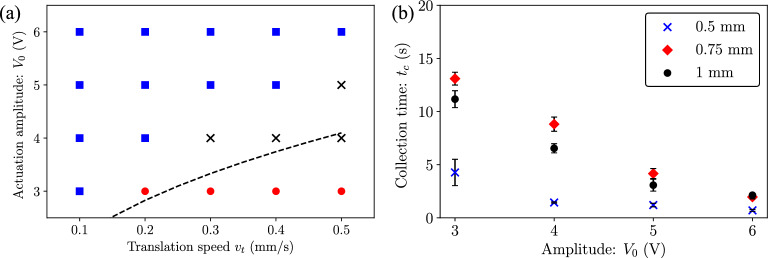
(**a**) Mapping showing the different collection states of particles at different actuation amplitudes ($V_0$) and translation speeds ($v_t$): stable collection between $\lambda /4$ and $\lambda /2$ (blue squares), unstable collection at $\lambda /4$ (black crosses) and dispersion (red circles). The dashed black line indicates the maximum tweezing speed estimates. (**b**) Collection times for glass particles ($r_p=$5 $\upmu \hbox {m}$) at different actuation amplitudes and liquid heights $h_l=$0.5, 0.75 and 1 $\hbox {mm}$.

As seen in Fig. 3b, when the capillary wave is displaced at a speed of $v_t=$0.1 $\hbox {mms}^{-1}$, the particles translate almost underneath the central node of the capillary wave at all times (Supplementary video SV3). When the translation speed is increased to $v_t=$0.5 $\hbox {mms}^{-1}$ (Fig. 3c), the particle displacement lags the displacement of the capillary wave and the particles translate at a distance $d_p$ away from the central node (indicated in Fig. 3a). As long as the particle position remains between $\lambda /4$ and $\lambda /2$ (or $d_p<\lambda /4$), the particles remain collected in a single line. When the actuation amplitude is decreased (Fig. 3d,e), particles can enter the region $0<x<\lambda /4$ and get drawn under the upper plate ($x=0$). The same phenomenon can be seen when the translation speed ($v_t$) is increased beyond 0.5 $\hbox {mms}^{-1}$. Figure 4a shows a mapping of the different particle collection states obtained at different actuation amplitudes ($V_0$) and translation speeds ($v_t$). It is clearly observed that as the translation speed increases or the actuation amplitude decreases, the tendency of particles to either collect in the unstable region of $x=\lambda /4$ (black crosses) or underneath the upper plates at $x=0$ (red squares) increases. Therefore, for each actuation amplitude there is a maximum tweezing speed where the particles will translate with the capillary wave, i.e., $d_p=\lambda /4$.

### Tweezing speed

An estimation of the maximum tweezing speed can be obtained by calculating the average collection speed of particles when the capillary wave is not undergoing translation. To ascertain the average collection speed, a collection time $t_c$ is identified for different actuation conditions by observing the pixel intensity over time near the collection region (details in Supplementary information Fig. 1).

Figure 4b shows the collection times $t_c$ for different actuation amplitudes and different liquid heights ($h_l$) at a frequency $f=$58 $\hbox {Hz}$. The particles collect faster with increasing actuation amplitude for all liquid heights. Considering that the displacement amplitude of the base ($A_0$) varies proportionally with the excitation voltage ($V_0$) at the same frequency, the collection time variation with actuation amplitude observed in experiments ($t_c \propto V_0^{-2.5}$) agrees closely with the scaling of $t_c\propto A_0^{-2}$ obtained from simulations. However, the variation of collection time with liquid height is not consistent. There are several factors which hinder a direct comparison of the variation of collection time with liquid height obtained from experiments with simulations. Capillary wave oscillation characteristics are similar to that of a forced damped oscillator, where the surface tension forces act as the spring restoring force and the viscosity of the liquid acts as a damper. In such a forced oscillation, there is a phase difference in the oscillation of the capillary wave velocity field and the oscillation source (this is the PDMS well in our experiments). More importantly, the capillary wave amplitude ($u_0$) will also vary with base actuation amplitude ($A_0$) around the resonant frequency (see Supplementary information Fig. 3 obtained via simulations). The relative value of these amplitudes is critical in determining particle movement speed as the flow field generated inside the liquid is a superposition of the capillary wave flow field and the Stokes boundary layer due to the oscillation of the bottom well. Considering a Stokes flow, the flow field in the *x*-direction at a height *z* from the base can be idealised as (Supplementary information Fig. 3)^61,62^:

3 $$\begin{aligned} u_1=A_0 \omega e^{(-\beta z)} \cos (\omega t-\beta z) +u_0 e^{-k (h_l-z)}\cos (kx-\omega t) , \end{aligned}$$

where the first term is the Stokes boundary and the second term is the capillary wave flow field layer. Here, $\beta =\sqrt{\omega /2\nu }$, is the Stokes boundary layer coefficient and $k=2\pi /\lambda$. Considering the capillary wave resonance frequency depends on the liquid height (Eq. 2), it is difficult to control and quantify the capillary wave amplitude across different liquid heights around their respective natural frequencies. Therefore, the collection time variations observed in experiments cannot be compared directly with simulation results. Considering the fastest collection is observed at $h_l=$ 0.5 $\hbox {mm}$, we use this height for all tweezing experiments.

To obtain an estimate of the maximum tweezing speed, we assume that the particles travel a distance of $\lambda /4$ in the time $t_c$ obtained from experiments. As a result, the theoretical average collection speed can be obtained as $v_{th}=\lambda /4t_c$. Figure 4a depicts the estimated maximum tweezing speed (black dashed line) as a function of the actuation amplitude. For any combination of actuation amplitude and translation speed lying above this dashed line, particles will translate with the capillary wave.

**Figure 5:**
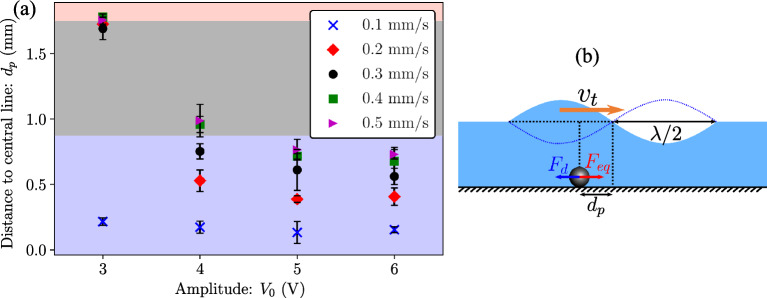
(**a**) Position of particles relative to the central wave node position ($d_p$) at different actuation amplitudes and translation speeds. The red, grey and blue regions represent the position $x=0$ (underneath the upper plates), $0<x<\lambda /4$ and $\lambda /4<x<\lambda /2$ (stable collection). (**b**) Depiction of $d_p$ under a capillary wave translating at a speed $v_t$. The figure also depicts the forces acting on the particles during tweezing; $F_d$ is the Stokes drag force due to capillary wave movement and $F_{eq}$ is the equivalent collection force time-averaged over a cycle due to the periodic inertial drag from the capillary wave^56^.

As observed in Fig. 3c,d, the movement of the collected particles lags the translation of the capillary wave at a distance $d_p$ away from the central node. Figure 5a shows the values of $d_p$ obtained via experiments for different actuation amplitudes and translation speeds. It can be seen that at the lowest amplitude ($V_0=$ 3 $\hbox {V}$), at all translation speeds apart from $v_t=$ $0.1\,\hbox {mms}^{-1}$, $d_p=$ 1.75 $\hbox {mm}$, which corresponds to the case where the particles have been drawn underneath the upper plates (red circles in Fig. 4a). It is to be noted that the points in the grey region in Fig. 5a indicate particles in the unstable collection region, which are indicated by black crosses in Fig. 4a. As the actuation amplitude is increased, particles start moving stably in the region $\lambda /4<x<\lambda /2$ (indicated by the blue shaded area in Fig. 5a). Similarly, a decrease in the translation speed decreases the distance between the central node of the wave and the particle movement.

This distance $d_p$ can be estimated by adjudging the balance of forces on the particles, as depicted in Fig. 5b. In the reference frame of the upper plates (which are undergoing a translation with a speed $v_t$), the particles experience an equivalent collection force ($F_{eq}$) from the periodic inertial drag due to the capillary wave, given by^56^:

4 $$\begin{aligned} F_{eq}=6\pi \mu r v_c + \frac{4}{3}\pi r^3 \rho _p a_c, \end{aligned}$$

where $v_c$ and $a_c$ are the average particle velocity and acceleration over a cycle, respectively, which are obtained from simulations (Supplementary information Fig. 5) and vary with the position of the particle relative to the node of the capillary wave ($v_c=0$ and $a_c=0$ at the collection position). The drag force ($F_d$) from the translation of the capillary wave, is given by the Stokes’ drag equation^61^:

5 $$\begin{aligned} F_{d}=6\pi \mu r v_t. \end{aligned}$$

As a simplistic approximation, the Eqs. (4) and (5) are obtained considering Stokes flow and do not consider the history and virtual mass forces^56^. The particles move with the capillary wave at a stable distance $d_p$ when the two forces are equal, i.e., $F_{eq}=F_{d}$. From simulations, $F_{eq}$ varies parabolically with the particle position, i.e., $F_{eq} =c_1x-c_0 x^2$, where $c_0$ and $c_1$ are scaling constants ($c_1$ and $c_0$ are obtained from simulations, details provided in supplementary information). Therefore, a relation between $v_t$ and $d_p$ can be written as:

6 $$\begin{aligned} 6\pi \mu r v_t=c_1d_p-c_0d_p^2. \end{aligned}$$

**Figure 6:**
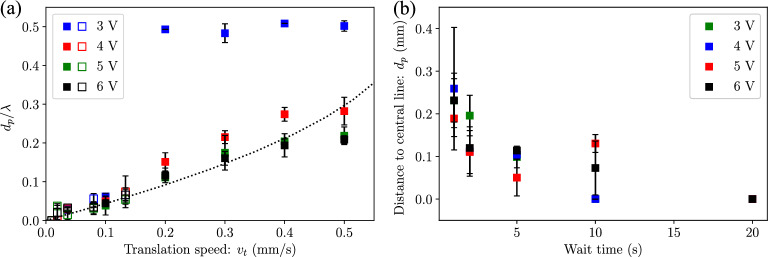
Distance of particle collection line from the center of the capillary wave during translation, normalised with the capillary wavelength ($d_p/\lambda$) at different capillary wave translation speeds ($v_t$) and excitation voltages ($V_0$). The closed markers represent the experiments for continuous translation and the open markers represent the experiments for stepped translation; $h_l$=0.5 $\hbox {mm}$, *d*=3.5 $\hbox {mm}$ (*f*=58 $\hbox {Hz}$). (**b**) Distance of particle collection line from the center of the capillary wave ($d_p$) during stepped translation of the capillary wave in steps of 0.2 $\hbox {mm}$ at speeds of 0.5 $\hbox {mms}^{-1}$. A wait time is introduced to allow the particles to move towards the collection region at this high translation speeds.

As shown in Fig. 6a, experimental data agrees well with the trend obtained from Eq. (6). To move particles at high translation speeds, while ensuring that particles remain collected in the region $\lambda /4<x<\lambda /2$, a stepped translation procedure can be implemented. In Fig. 6b, the capillary wave is moved a distance of 0.2 $\hbox {mm}$ at a velocity of $v_t=$ 0.5 $\hbox {mms}^{-1}$ and held stationary for a fixed waiting time before initiating translation again. This waiting time allows the particles to move towards the central node position before the capillary wave undergoes another displacement. Figure 6b shows the value of $d_p$ at the velocity $v_t=$ 0.5 $\hbox {mms}^{-1}$ at different waiting times of 1, 2, 5, 10 and 20 s. At the longest wait time of 20 s for all amplitudes and plate velocities the distance to the central line is always 0, indicating that the collected particle line consistently returns to the central point. Therefore, the introduction of this waiting time reduces the average translation velocity of the capillary wave. When overlayed with the data from the continuous translation, Fig. 6a shows an agreement of $d_p$ with these average velocities obtained from the numerical model.

### Tweezer geometry

**Figure 7:**
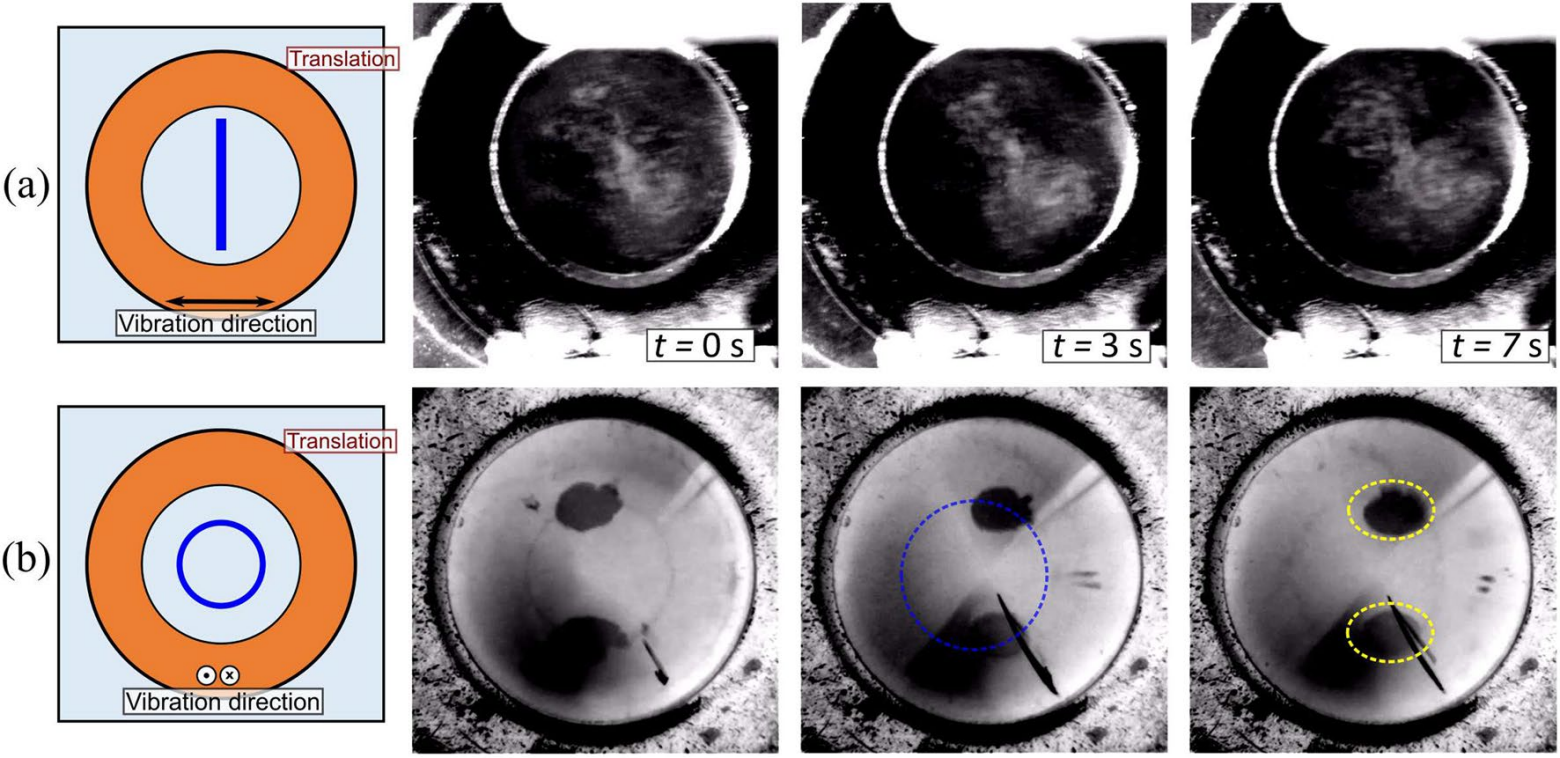
(**a**) Collection of $r_p=$10 $\upmu \hbox {m}$ glass particles through a capillary wave excited in a ring (diameter 6 $\hbox {mm}$); the base here is vibrated horizontally at $V_0=$1 $\hbox {V}$ and a frequency of 24 $\hbox {Hz}$, which sets up a wave of one wavelength, as indicated in the inset. (**b**) Collection of $r_p=$10 $\upmu \hbox {m}$ glass particles through a capillary wave excited in a ring (diameter 8 $\hbox {mm}$); the base here is vibrated vertically at $V_0=$ ${3}\,\hbox {V}$ and frequency of 35 $\hbox {Hz}$, which sets up a wave of 1.5 wavelength, as indicated in the inset. The yellow dashed lines indicate particles trapped at the water-air interface. The images in (**a**,**b**) have been altered digitally by inversion and contrast adjustment for clarity.

In the previous experiments, capillary waves were obtained by actuating the base. These waves can also be excited by actuating the upper plates across which the waves are generated. Additionally, by altering the geometry of the pinned contact line, the vibration modes of the liquid-air interface can be altered, which influences the particle collection pattern. In Fig. 7a,b, a circular ring geometry (a washer) is used to generate a wave with a circular pinned contact line. The waves produced in this configuration are similar to that in a vibrating droplet with a pinned contact line, which demonstrates symmetric and asymmetric waves depending on the actuation frequency and direction of vibration^66^. For example, in Fig. 7a (Supplementary video SV5), when the washer is vibrated horizontally at 24 $\hbox {Hz}$ a wave of 1 wavelength is formed; theoretical frequency for a 1 wavelength wave is 20 $\hbox {Hz}$ from Eq. (2). As in the previous cases with a straight contact line, the particles collect underneath the displacement nodes of the capillary wave. When the base well containing the liquid undergoes translation (from right to left), the particles remain trapped underneath the displacement node of the capillary wave. When the washer is vibrated vertically, axially symmetric waves can be generated. In Fig. 7b (and Supplementary video SV5) a wave of 1.5 wavelength is generated at a frequency of 35 $\hbox {Hz}$; theoretical frequency for a 1.5 wavelength wave is 42 $\hbox {Hz}$ from Eq. (2). As the liquid-air interface oscillation is axially symmetric the particle collection pattern forms a ring of diameter 4.1 $\hbox {mm}$, as indicated by the blue dotted line in Fig. 7b. Under such vertical vibration conditions, particles are observed to collect underneath the displacement antinodes of the capillary wave, e.g. in sessile droplets^67^ and rectangular chambers^57^. Similar to the observations in Fig. 7a, when the base liquid well undergoes translation (from right to left) the particle ring remains trapped underneath the displacement antinode. In addition to the ring near the base, some particles are also seen to collect in two clusters (indicated by the yellow dashed lines in Fig. 7b. These particles are located at the water-air interface and get trapped in the nodal locations of the capillary wave^68^.

## Conclusion

In this work, we presented a new concept of using capillary waves as tweezers to control the movement of microparticles. Capillary waves are generated in an open liquid volume between a set of movable solid boundaries. The capillary wave induces a stable collection of microparticles in the liquid volume in a line underneath the displacement node of the capillary wave. As the boundaries are displaced, the capillary wave field drags the particles with it, where the particle movement lags the movement of the capillary wave. Using analytical and numerical models we predict the conditions in which a stable continuous movement of particles is obtained. Finally, we demonstrate that different tweezer designs can also be used to trap and move particles by using a ring-shaped boundary. The concept of using interfacial wave based tweezers allow the potential for a programmable control of particle motion in open microfluidic systems. For example, controllable capillary waves can be used for non-contact movement, positioning and probing cells for 3D bio-printing. Such programmable tweezers can also be used to assemble particles in defined patterns for micro-manufacturing and surface coatings. As the wavelength of capillary waves can also be scaled up (by lowering the frequency), such programmable systems can also be used for large scale separation of microplastics from wastewater streams.

## Methods

### Experimental setup

Monodisperse 10 $\upmu \hbox {m}$ melamine resin based microparticles and 10 $\upmu \hbox {m}$ glass particles (Sigma Aldrich) were used for the experiments. The particles were obtained in 10% v/v solution and were diluted to 1% by adding DI water. The particle solutions were sonicated for 5 minutes before use to avoid agglomeration. A chamber of dimensions length (*L*) 8 $\hbox {mm}$, width (*W*) 4 $\hbox {mm}$ and depth ($h_w$) 200 $\upmu \hbox {m}$ made from PDMS was used to contain the water-particle mixture. Vibrations were provided using a permanent magnet shaker (Brüel and Kjaer, LDS V201) connected to a signal generator (Farnell, Sine Squared Oscillator LF1). The upper plates were fabricated from PDMS mounted on acrylic sheets. The horizontal translation motion of the upper plates was controlled using a motorized linear stage (Thor Labs MTS50/M-Z8) connected to a controller (Kinesis cube KDC101). The height of the liquid volume was altered by mounting the upper plates on a manual *z*-translation stage (Thor Labs PT1/M). Particle motion was recorded using a camera (IDS UI 3070SE-C-HQ) and a Navitar 12x zoom lens. Simulation details and methodology are provided in the supplementary information.

### Collection time characterisation

Particle collection time is characterised by observing the pixel intensity profile across a line in the videos (Supplementary Fig. 1a). The pixel intensity near the collection location (underneath the central displacement node) increases over time. This maximum pixel intensity is tracked over time (Supplementary Fig. 1b). The collection time ($t_c$) is identified as the earliest time instance when the change in pixel intensity is less than 10%.

## Supplementary Information

Supplementary Information 1.

Supplementary Information 2.

Supplementary Information 3.

Supplementary Information 4.

Supplementary Information 5.

Supplementary Information 6.

## Data Availability

The authors declare that majority of the data supporting the findings of this study are available within the paper and its supplementary information files. More detailed specific data is available from the corresponding author on request.
