# Supplementary material for: Capillary wave tweezer
Source: Sci Rep. 2024 May 30;14:12448. doi: 10.1038/s41598-024-63154-0 (PMC11637047; doi:10.1038/s41598-024-63154-0)
Supplement: Supplementary file 1 — Supplementary Information 1. [file 41598_2024_63154_MOESM1_ESM.pdf]

Supplementary information: Capillary wave tweezers

Bethany Orme[1], Hamdi Torun[1], Matthew Unthank[2], Yong-Qing Fu[1], Bethan Ford[1], and Prashant Agrawal[1*]

[1]Smart Materials and Surfaces Laboratory, Faculty of Engineering and Environment, Northumbria University, Newcastle upon Tyne NE1 8ST, United Kingdom
[2]Department of Applied Sciences, Northumbria University, Newcastle upon Tyne NE1 8ST, United Kingdom
Corresponding author: prashant.agrawal@northumbria.ac.uk

1 Particle collection time characterisation

Particle collection time is characterised by observing the pixel intensity profile across a line in the videos (Fig. 1 (a)). The pixel intensity near the collection location (underneath the central displacement node) increases over time. This maximum pixel intensity is tracked over time (Fig. 1 (b)). The collection time (t_c) is identified as the earliest time instance when the change in pixel intensity is less than 10%.

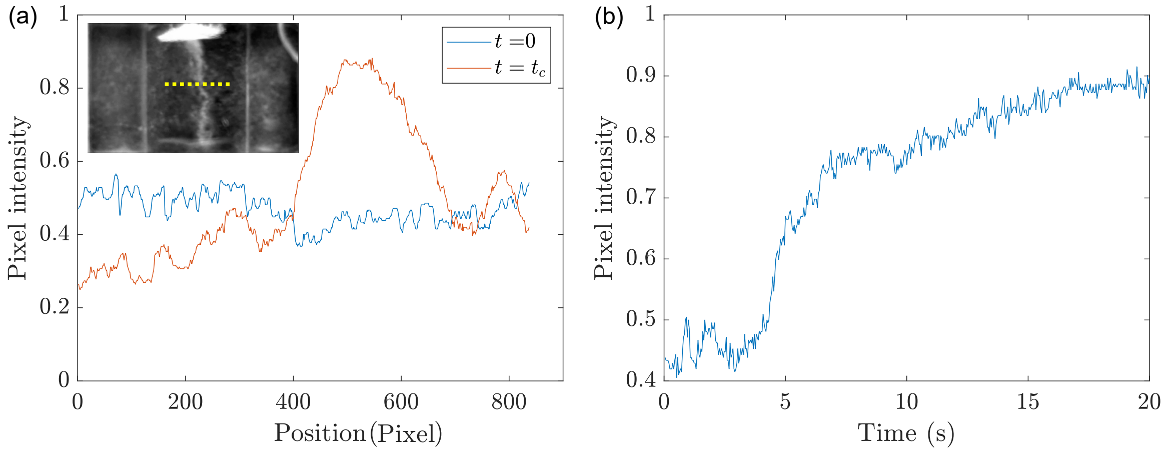

Figure 1: (a) Change in pixel intensity across a line (dashed yellow) before the start of actuation ($t = 0$) and after collection ($t = t_c$); (b) Variation of maximum pixel intensity across the line with time.

1.1 Simulation Methodology

The simulation setup consists of three components: 1) first order periodic flow field, 2) time-averaged second-order flow field and 3) particle tracing algorithm. COMSOL multiphysics is used to simulate the first and time-averaged second order flow fields, while particle tracing is performed in MATLAB. The first order and second order flow field components can be obtained using perturbation theory on the Navier Stokes equation[1]. Here the response of a system under a perturbation (ε) from a state of equilibrium can be represented by a superposition of different orders of the flow field parameters, the pressure (p) and velocity fields ($\mathbf{u}$), and can be written as:

$$\mathbf{u} = \mathbf{u}_0 + \varepsilon \mathbf{u}_1 + \varepsilon^2 \mathbf{u}_2 + ..., \quad (1)$$

$$p = p_0 + \varepsilon p_1 + \varepsilon^2 p_2 + ... \quad (2)$$

Here, the terms with the coefficient ε and ε^2 represent the first and second order flow fields, respectively. The first and second order components are also denoted by the subscripts. p_0 is equal to the atmospheric pressure and $\mathbf{u}_0 = 0$ considering an initial state of rest. Inserting equations 1 and 2 into the Navier-Stokes equations 3a and 3b,

$$\rho_0(\nabla \cdot \mathbf{u}) = 0, \tag{3a}$$

$$\rho_0\left(\frac{\partial \mathbf{u}}{\partial t} + \mathbf{u}\cdot(\nabla \mathbf{u})\right) = -\nabla p + \mu \nabla^2 \mathbf{u} + \mathbf{f}. \tag{3b}$$

and collecting the coefficients of ε and ε^2 results in the first order flow field equations,

$$\rho_0(\nabla \cdot \mathbf{u}_1) = 0, \tag{4a}$$

$$\rho_0 \frac{\partial \mathbf{u}_1}{\partial t} = -\nabla p_1 + \mu \nabla^2 \mathbf{u}_1 + \mathbf{f}. \tag{4b}$$

and the second-order flow field equations,

$$\nabla \cdot \mathbf{u}_2 = 0, \tag{5a}$$

$$\rho_0 \frac{\partial \mathbf{u}_2}{\partial t} + \mu \nabla^2 \mathbf{u}_2 = \nabla p_2 + \rho_0[(\mathbf{u}_1 \cdot \nabla)\mathbf{u}_1 + \mathbf{u}_1(\nabla \cdot \mathbf{u}_1)]. \tag{5b}$$

Here, ρ_0 and μ are the density and dynamic viscosity of the liquid, respectively and $\mathbf{f}$ the external body force acting on the liquid volume.

1.1.1 Vibration induced first order flow field

The simulated liquid domain with the boundary conditions for the first order flow field is shown in Fig. 2 (a). To incorporate surface tension, the normal stress condition at the liquid-air interface is implemented as:

$$\left[-p_1\mathbf{I} + \mu(\nabla \mathbf{u}_1 + (\nabla \mathbf{u}_1)^T)\right] \cdot \mathbf{n} = \Gamma \gamma \mathbf{n}, \tag{6}$$

where, Γ is the liquid-air interface curvature, γ is the surface tension of water, $\mathbf{I}$ is a unit matrix and $\mathbf{n}$ is the unit normal vector. Starting form rest, transient simulations are performed for about 25 cycles to ensure a periodic field has been set up. For calculating the time-averaged second order fields, the data of the last cycle is extracted and fed into the next simulation step.

1.1.2 Time-averaged second-order flow field

The second order flow equation 5b show that the velocity gradients of the first order field act as a body force to drive the second order flows. Considering linear actuation conditions, i.e., the actuation amplitude is significantly smaller than the wavelength ($A_0/\lambda << 1$), the instantaneous magnitude of the second order flow field will be negligible compared to the first order flow field magnitude. However, time-averaged effects can be significant, as observed in acoustic and sub-acoustic systems[2, 3, 4]. Therefore, the time-averaged second order flow field equations are obtained as:

$$\nabla \cdot \langle \mathbf{u}_2 \rangle = 0, \tag{7a}$$

$$\mu \nabla^2 \langle \mathbf{u}_2 \rangle = \nabla \langle p_2 \rangle - \mathbf{F}. \tag{7b}$$

Here, $\langle x \rangle$ is the time-averaged value of the variable x and,

$$\mathbf{F} = -\rho_0 \langle (\mathbf{u}_1 \cdot \nabla)\mathbf{u}_1 + \mathbf{u}_1(\nabla \cdot \mathbf{u}_1) \rangle. \tag{8}$$

The simulation boundary conditions on the liquid domain for the time-averaged flow fields are shown in Fig. 2 (b). The final velocity field acting on the particles is obtained as:

$$\mathbf{u} = \mathbf{u}_1 + \langle \mathbf{u}_2 \rangle. \tag{9}$$

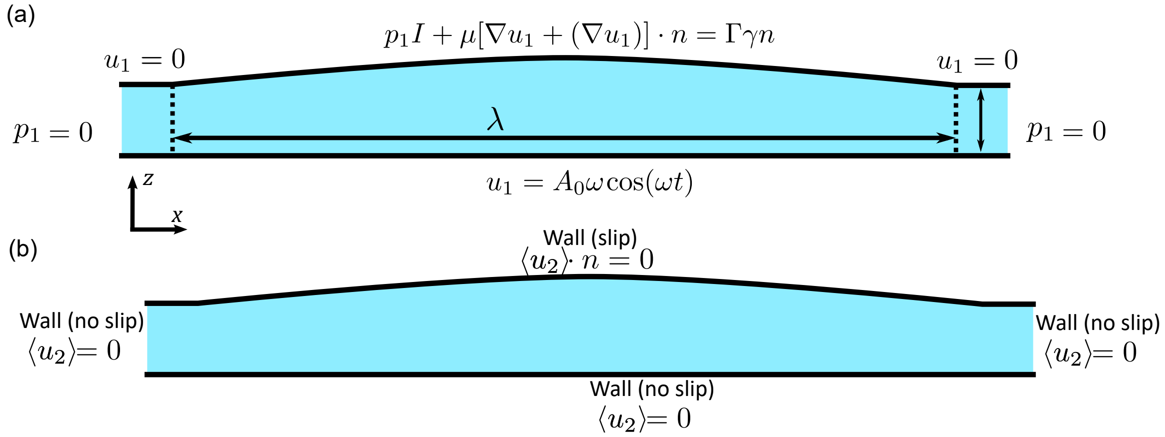

Figure 2: Simulation domain and boundary conditions to simulate: (a) First order field, (b) Time-averaged second order field.

1.1.3 Particle Tracing Algorithm

Particle motion simulation is performed by extracting the flow fields from COMSOL and importing in MATLAB. Bi-linear interpolation is performed on the spatial and temporal velocity grid data to increase the data resolution. Particle motion is simulated using the equation,

$$m_p \ddot{\mathbf{x}} = \mathbf{F}_p, \tag{10}$$

where, m_p is the mass of the particle, $\mathbf{x}$ is the displacement vector and $\mathbf{F}_p$ is the drag force on the particle, given by[5]:

$$\mathbf{F}_v = \pi r^2 \rho_0 |\mathbf{u} - \mathbf{u}_p|(\mathbf{u} - \mathbf{u}_p)[1.84 Re_p^{-0.31} + 0.293 Re_p^{0.06}]^{3.45}. \tag{11}$$

Here, $Re_p = 2\rho_p \mathbf{u}_p r/\mu$ is the particle Reynold's number, r and ρ_p are the particle's radius and density, respectively and $\mathbf{u}_P$ is the particle's velocity vector. A second order finite difference scheme is used to discretise the equation 10.

2 Effect of liquid depth on collection time

In our system, where the substrate is vibrated horizontally (in x-direction), the flow field generated inside the liquid is a superposition of the capillary wave flow field and the Stokes boundary layer due to the oscillation of the bottom well. Considering a Stokes flow, the flow field in the x-direction can be idealised as:

$$u_1 = A_0 \omega e^{(-\beta z)} \cos(\omega t - \beta z) + u_0 e^{-k(h-z)} \cos(kx - \omega t), \tag{12}$$

where the first term is the Stokes boundary and the second term is the capillary wave flow field layer[6, 7]. Here, $\beta = \sqrt{\omega/2\nu}$, is the Stokes boundary layer coefficient and $k = 2\pi/\lambda$. The flow field map of equation 12 is depicted in Fig. 3 (a). Here, two key factors affect the strength of the gradient field experienced by a particle at a height $z = r$ from the base, which eventually affects its collection time: 1) penetration depth of the capillary wave and 2) the capillary wave field magnitude u_0 at $z = r$.

2.1 Penetration depth of capillary wave field

Based on the second term, the penetration depth of the capillary wave flow field is λ, i.e., the magnitude of the capillary wave flow field will decrease by 90% at a depth of $2.3/k$ (or 0.37λ) from the liquid-air interface. Therefore, the penetration depth of this capillary wave field depends on the actuation frequency. As the capillary wave field generates the spatial gradients resulting in a net particle movement towards the collection location, the gradients experienced by particles at a certain height z, will depend significantly on the actuation frequency, which will eventually affect their collection time.

2.2 Damped oscillation of the capillary wave

Figure 3 (b) shows the amplitude response of the capillary wave around the resonant frequency obtained from simulations. The capillary wave oscillations demonstrate a damped oscillation behaviour, where the surface tension acts as the restoring force and the viscosity acts as the damper. For a fixed actuation amplitude of the base (A_0), the capillary wave amplitude u_0 will be maximum at the resonant frequency of the wave and will be damped at frequencies around it. As the wave frequency changes with the liquid depth, actuating the base away from the natural frequency will lead to a damped response of the capillary wave amplitude, which affects the magnitude of gradients experienced by the particle and influences their collection time.

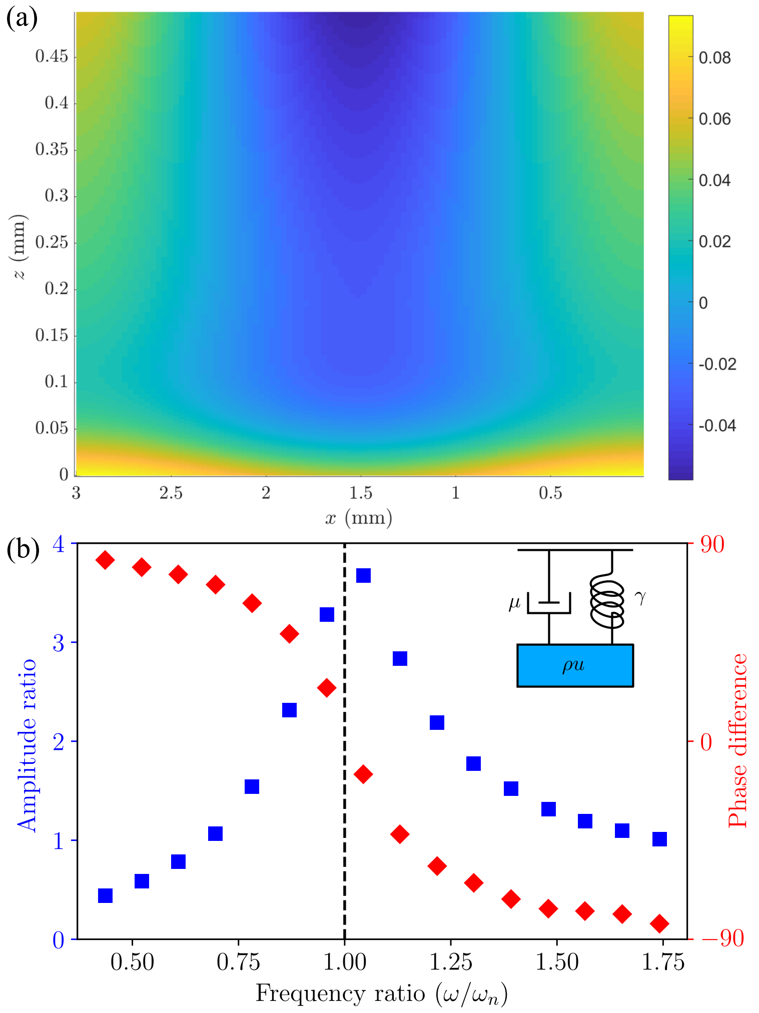

Figure 3: (a) Example of the superimposed Stokes boundary layer flow field and the capillary wave flow field; the color bar on the right represenst the flow field magnitude. (b) Phase difference and amplitude ratio ($u_0/A_0\omega$) of the velocity field with the source excitation. The damped response is similar to that of a spring-mass-damper system where the surface tension acts as a spring and viscosity acts as a damper.

3 Particle motion in spatially varying flow fields

Figure 4 (a) shows the motion of a solid particle (red) and a fluid particle (blue) in a uniform and in a spatially varying flow field over 1 oscillation cycle. In a uniform flow field, the particle moves with a phase and amplitude lag, but returns to its original position after 1 cycle. In a spatially varying flow field (magnitude decreases from left to right), the particle experiences a stronger flow field when it is on the left of the fluid particle, and a weaker flow field on the right of the fluid particle, at different times in the cycle. This difference in fluid flow field and, therefore, the drag force, results in a net displacement of the particle after 1 cycle [5].

Figure 4 (b) shows the motion of particles at different positions across a wavelength underneath a capillary wave. The positions $x = 0$, $\lambda/2$ and λ are stable regions where particles converge because the superposition of the capillary wave field and the Stokes flow field results in positions underneath the capillary wave to have the lowest velocity field magnitude (Fig. 3 (a)). The positions $x = \lambda/4$ and $3\lambda/4$ are unstable regions from where particles diverge.

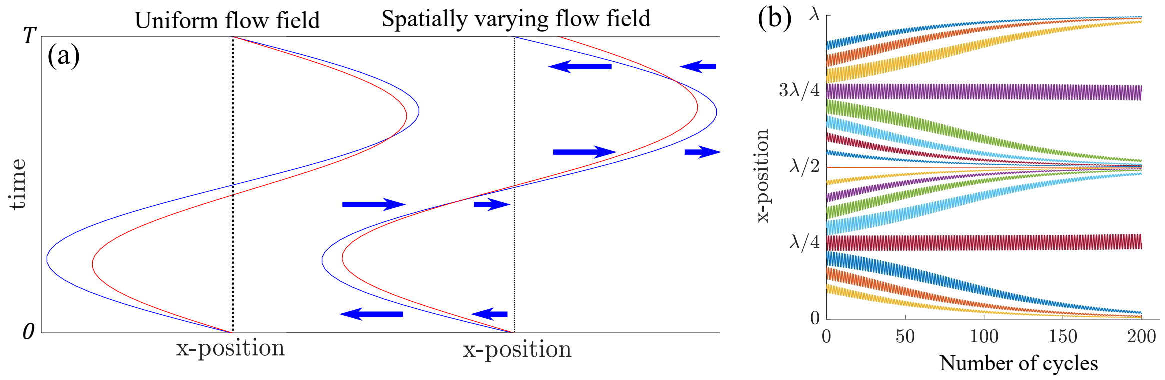

Figure 4: (a) Movement of a solid particle (red) and a fluid particle (blue) in a uniform and in a spatially varying flow field over one cycle with time period T at a given position. The blue arrows depict the strength of the flow field on either side of the fluid particle motion, i.e., the flow field magnitude decreases from left to right; (b) Motion of particles starting at different positions across a wavelength underneath a capillary wave.

4 Equivalent collection force

The particle undergoes a net displacement towards the collection location after each cycle under the drag force from a spatially and temporally varying flow field (Fig. 5). To represent the time-averaged motion of the particle we define an equivalent collection force F_{eq}. The expression for F_{eq} is obtained in an equivalent system where the liquid is at rest and a force F_{eq} acts on the particle imparting it an instantaneous velocity v_c. In such an equivalent system, the acceleration of the particle a_c can be written as[5]:

$$ma_c = F_{eq} - 6\pi\mu r v_c, \tag{13}$$

which can be re-arranged to give

$$F_{eq} = 6\pi\mu r v_c + ma_c, \tag{14}$$

where, m is the mass of the particle, μ is the liquid viscosity and v_c and a_c are the time-averaged velocity and acceleration of the particle, given by:

$$v_c = \frac{x_{t+T} - x_{t-T}}{2T}, \tag{15}$$

$$a_c = \frac{x_{t+T} - 2x_t + x_{t-T}}{T^2}. \tag{16}$$

Here, x_{t+T}, x_t and x_{t-T} are the position of the particles after each cycle with a time-period $T = 2\pi/\omega$ as depicted in Fig. 5 (c).

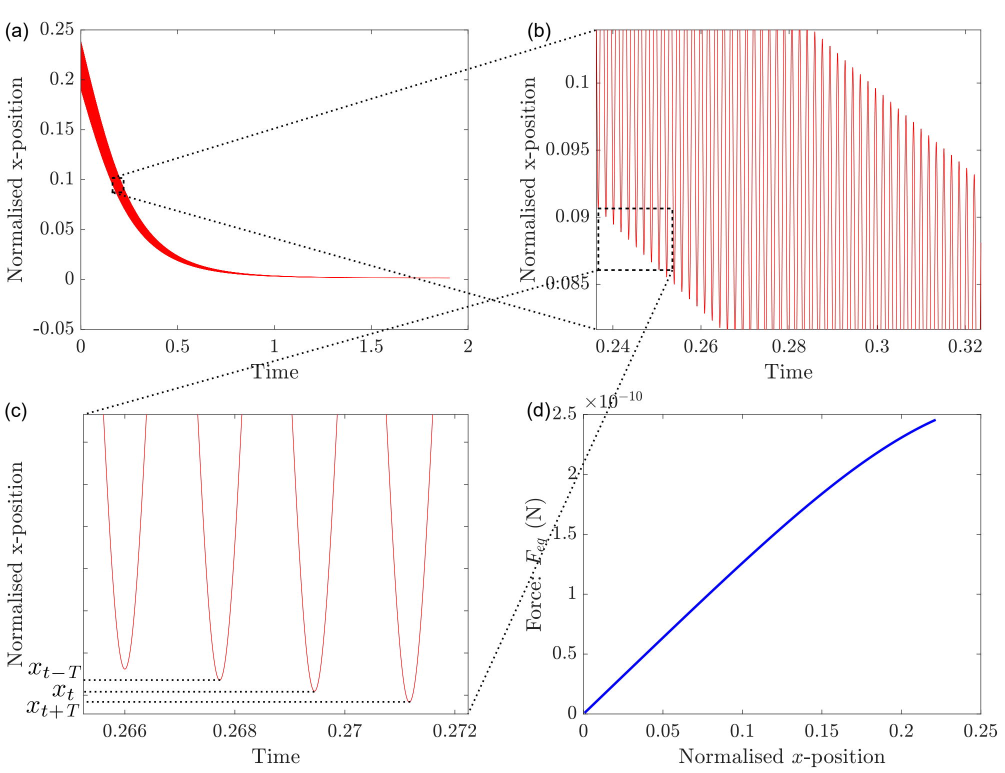

Figure 5: (a) Position of a 10 μm diameter particle in the flow field obtained from simulations over time, (b) exploded view of the particle movement, (c) particle movement over a few cycles indicating the extraction of particle positions to calculate the equivalent force, (d) equivalent force on the particle at different positions normalised with the wavelength.

The expression for F_{eq} obtained from the Figure 5 (d) using a second order polynomial fit on the normalised x-position ($\bar{x} = 0.5 - x/\lambda$)is:

$$F_{eq} = \left(13\bar{x} - 9.9\bar{x}^2\right) \times 10^{-10} \tag{17}$$

References

[1] Henrik Bruus. Acoustofluidics 2: Perturbation theory and ultrasound resonance modes. *Lab Chip*, 12:20–28, 2012.

[2] S. M. Hagsäter, T. Glasdam Jensen, H. Bruus, and J. P. Kutter. Acoustic resonances in microfluidic chips: full-image micro-PIV experiments and numerical simulations. *Lab Chip*, 7:1336–1344, 2007.

[3] Kenneth D. Frampton, Shawn E. Martin, and Keith Minor. The scaling of acoustic streaming for application in micro-fluidic devices. *Applied Acoustics*, 64(7):681 – 692, 2003.

[4] D. Klotsa, Michael R. Swift, R. M. Bowley, and P. J. King. Interaction of spheres in oscillatory fluid flows. *Phys. Rev. E*, 76:056314, 2007.

[5] P. Agrawal, P.S. Gandhi, and A. Neild. Microparticle response to two-dimensional streaming flows in rectangular chambers undergoing low-frequency horizontal vibrations. *Physical Review Applied*, 2:064008, 2014.

[6] L D Landau and E M Lifshitz. Fluid Mechanics, 1987.

[7] Prashant Agrawal, Prasanna S. P.S. Gandhi, and Adrian Neild. The mechanics of microparticle collection in an open fluid volume undergoing low frequency horizontal vibration. *Journal of Applied Physics*, 114:114904, 2013.
